# Comparative assessment of the spot sign and leakage sign as predictive factors for spontaneous intracranial hematoma expansion

**DOI:** 10.1007/s10140-025-02352-3

**Published:** 2025-06-12

**Authors:** María Del Carmen González Domínguez, Roberto Fornell-Pérez, Ernesto Santana Suárez, Diego Riol Sancho, Elisabet González Domínguez, Juan Francisco Loro-Ferrer

**Affiliations:** 1 Department of Clinical Sciences, University of Las Palmas de Gran Canaria. Las Palmas. Spain. Complejo Hospitalario Universitario Insular Materno-Infantil de Las Palmas de Gran Canaria, Las Palmas, Spain; 2 Hospital Universitario Basurto, Bilbao, Spain; 3 Complejo Hospitalario Universitario Insular Materno-Infantil de Las Palmas de Gran Canaria, Las Palmas, Spain; 4 Hospital Nuestra Señora de Guadalupe. San Sebastián de La Gomera, Santa Cruz, Spain; 5 Department of Clinical Sciences, University of Las Palmas de Gran Canaria, Las Palmas, Spain

**Keywords:** Acute stroke, Intracranial hemorrhages, Computed tomography, Inhospital mortality, Neurological deterioration, Predictive value of tests

## Abstract

**Objectives:**

To evaluate the predictive value of two radiological markers, the spot sign and leakage sign, for spontaneous intracranial hematoma expansion and their association with clinical outcomes, including neurological deterioration and in-hospital mortality.

**Materials & methods:**

This prospective single-center study included 94 adult patients with spontaneous intraparenchymal hemorrhagic stroke, confirmed by non-enhanced CT (NECT) and contrast-enhanced CT (CECT) in the arterial phase. Hematoma volumes and spot/leakage signs were assessed using standardized imaging protocols and analyzed by two blinded neuroradiologists. Clinical and radiological data were evaluated using multivariate analyses, with survival outcomes compared via Kaplan–Meier curves. Statistical significance was set at p ≤ 0.05.

**Results:**

Among 94 patients, hematoma expansion occurred in 42%, neurological deterioration in 15.5%, and mortality in 39.4%. The leakage sign was the strongest independent predictor of hematoma expansion (OR: 9.27, 95% CI: 2.95–29.20), neurological deterioration (OR: 26.67, 95% CI: 1.62–47.39), and mortality (OR: 7.56, 95% CI: 2.97–19.25). The spot and leakage signs demonstrated high specificity for predicting outcomes, with the leakage sign showing greater sensitivity for hematoma expansion. Patients with a positive leakage sign had significantly lower median survival (6 days) compared to those with a positive spot sign alone (54 days) or no signs (110 days, p < 0.001).

**Conclusion:**

The leakage sign demonstrated greater sensitivity and comparable specificity to the spot sign for predicting hematoma expansion. Both signs were associated with neurological deterioration and in-hospital mortality, with the leakage sign showing a stronger predictive value.

**Graphical Abstract:**

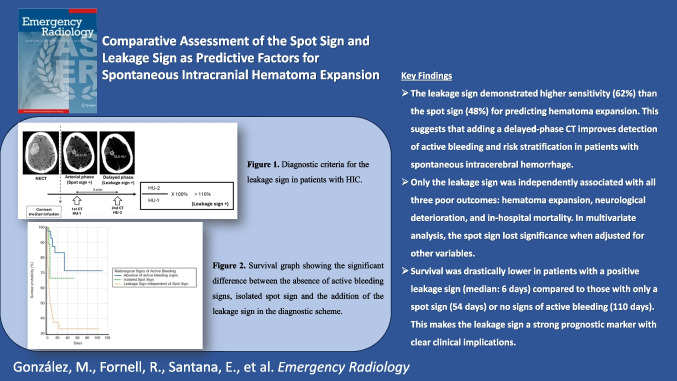

## Introduction

Hemorrhagic stroke is a condition with high morbidity and mortality rates, accounting for 10% to 20% of all strokes [1]. Intracerebral hemorrhage (ICH) involves the rupture of cerebral blood vessels with subsequent diffusion of blood into the brain parenchyma [2]. ICH can be classified as primary (spontaneous) or secondary (usually traumatic), with the former having an incidence of approximately 10 to 30 per 100,000 inhabitants [3]. The global incidence of spontaneous ICH has progressively increased over the years, a trend attributed to the growing prevalence of hypertension in the population [4]. Common risk factors for ICH include chronic hypertension, amyloid angiopathy, anticoagulant medication, and vascular malformations [5].

The volume of ICH serves as a useful parameter in predicting patient mortality [6]. Several studies have explored the relationship between hematoma expansion in spontaneous ICH and its impact on patient outcomes, including hospitalization costs. Hematoma expansion occurs in up to 30% of ICH patients, often within the first 24 h, and is associated with worse neurological outcomes, higher mortality, and increased healthcare costs [7–9].

Accurately predicting the expansion of spontaneous intracranial hematomas remains a challenge [10]. Some signs, based on the morphology and density of the hematomas in non-contrast-enhanced computed tomography (NECT), have been described as predictive of ICH growth [11], including the blend sign [12, 13], black hole sign [7], satellite sign [14], and island sign [15].

The aim of this study was to evaluate the differences between the spot sign and the leakage sign as predictive factors of spontaneous intracranial hematoma expansion. A secondary objective was to assess their relationship with poor neurological outcomes and mortality. These signs have been identified in previous studies as potential indicators of active bleeding within the brain parenchyma. Both are important predictors because their presence is associated with a higher likelihood of hematoma expansion, which can lead to increased intracranial pressure, accelerated neurological deterioration, and a higher risk of mortality. Studying these signs is crucial for improving our ability to predict and potentially intervene in the progression of intracranial hemorrhages [16].

## Materials and methods

### Study Design and Patients

This was a prospective, single-center longitudinal study of adult patients who presented to our hospital's emergency department between May 2017 and January 2020 with a diagnosis of spontaneous intraparenchymal hemorrhagic stroke.

Inclusion criteria were: a) patients over 18 years old; b) clinical presentation of cerebral stroke; c) final diagnosis of spontaneous intraparenchymal hemorrhagic stroke; d) NECT performed during the emergency episode, and e) contrast-enhanced computed tomography (CECT) in the late arterial phase to assess spot/leakage signs.

Exclusion criteria were: 1) intracranial bleeding secondary to other processes (e.g., trauma, ruptured cerebral aneurysms, arteriovenous malformations, hemorrhagic transformation of ischemic stroke, venous infarcts, or tumors); 2) technically suboptimal imaging studies due to movement artifacts or inadequate contrast enhancement; 3) lack of contrast administration due to severe renal insufficiency or confirmed allergy; 4) loss to follow-up due to patient transfer to other hospitals.

Patients who met the inclusion criteria were consecutively included until the final sample size of 94 patients was reached. The CT protocol used for the study was approved by the hospital's ethics committee.

### Informed Consent

Informed consent was waived due to the emergency nature of the clinical condition of most patients.

### Imaging Acquisition

All studies were performed using a 64-detector CT scanner (Somatom Definition Edge, Siemens Healthineers, Germany). According to the standardized protocol at our center, all patients with suspected ischemic-hemorrhagic pathology routinely underwent NECT from the cranial vertex to the skull base (section thickness 2.5 mm) at 120 kVp and 320 mAs. In cases where spontaneous ICH was observed, the study was followed by CECT after the administration of contrast medium (70 mL of 300 mg/mL iopromide) in the arterial phase (35 s post-injection) at a flow rate of 5 mL/s via a peripheral antecubital vein using a contrast injector, with the following parameters: 120 kVp, 240 mAs, section thickness of 2.5 mm, section acquisition interval of 1.25 mm, and pitch of 0.875:1. A third helical CT was acquired in the late phase (5 min post-injection of contrast) using the same technical parameters.

In most cases, a follow-up CT was performed 24 h after the initial diagnosis using the same parameters, to evaluate hematoma size and other intracranial findings. If there was evident neurological deterioration, a follow-up CT was performed sooner based on clinical judgment. A total of 25 patients did not undergo follow-up CT due to early death or clinical issues unrelated to the hemorrhagic stroke.

### Demographic and Clinical Data

Demographic and clinical variables were collected from patient records, including age, sex, systolic and diastolic blood pressure, prior treatment with antiplatelet or anticoagulant therapy, platelet count, INR, aPTT, and scores on the Glasgow Coma Scale (GCS) and National Institutes of Health Stroke Scale (NIHSS). Neurological deterioration was defined as an increase of at least 4 points on the NIHSS from the patient's baseline at the time of diagnosis [17]. In cases of in-hospital death, the cause was recorded as stroke-related or unrelated.

### Radiological Data

At the time of diagnosis, the location and volume of intraparenchymal hematomas were assessed on NECT images. Hematoma volume was also recorded on follow-up CT, which was routinely performed 24 h after diagnosis. In our study, significant hematoma expansion was defined as a volume increase of more than 15% on follow-up CT compared to the initial NECT. This threshold was used as a reference for semi-automated volume calculation using Syngo.via software (Siemens Healthcare), which reduces measurement variability. This was compared with previous studies, where the threshold typically ranged between 10 and 30%.

### Criteria for Detection of Spot Sign and Leakage Sign

Before the study began, a protocol was established for patients with suspected spontaneous ICH on their initial NECT, in which they underwent a complete imaging study. All imaging studies were reviewed by two neuroradiologists (with more than 5 years of experience) who were blinded to the patients’ clinical data and previous reports. These radiologists performed a consensus reading of each case, in two separate phases:

1. In the first phase, the radiologists reviewed only the arterial-phase CECT to determine the presence of the spot sign, without access to the late-phase images.
2. In the second phase, after at least 15 days, the radiologists reviewed both the arterial- and late-phase CECT to assess for the leakage sign.

The location and volume of the hematomas were assessed on the initial NECT, as well as on the follow-up NECT at 24 h (or earlier in cases of clinical deterioration). The hematoma volume was calculated using the semi-automated volume measurement tool included in the Syngo.via software (Siemens Healthineers, Germany).

To determine the presence of the spot sign, arterial-phase CECT images were visually inspected for any areas of increased density compared to the rest of the hematoma or compared to the initial NECT, excluding intracranial vascular structures or components of the hemorrhage itself.

**Fig. 1:**
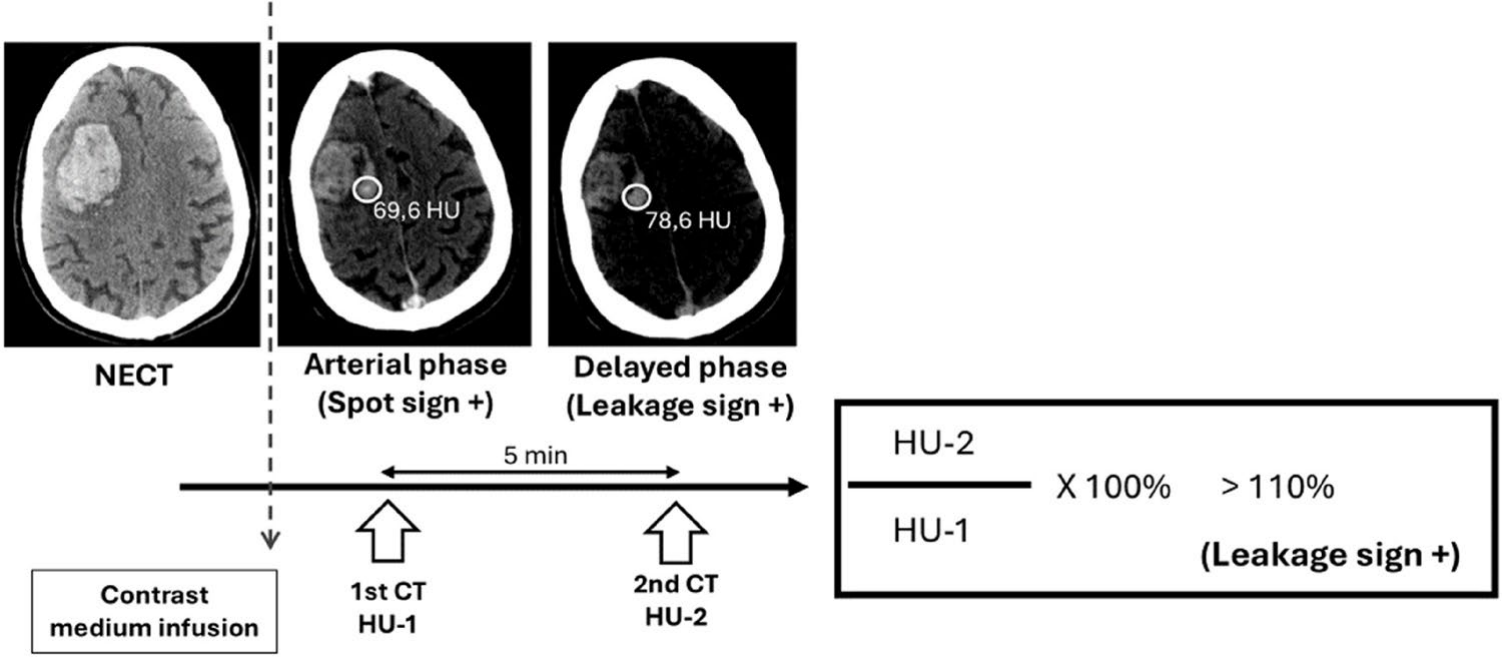
Diagnostic criteria for the leakage sign in patients with HIC. Based on the arterial phase CT and delayed phase CT images, a 1-cm-diameter region of interest (ROI) was set on the delayed phase images for the leakage of the contrast medium into the hematoma; the Hounsfield unit (HU) values in the ROI were determined in each section of the arterial and delayed phase images; and a > 10% increase in HU was considered a positive leakage sign

For the leakage sign, a similar process was followed, but using the late-phase CECT. Observers then placed a second 10-mm ROI on the arterial-phase images at the same anatomical location as the ROI on the late-phase images. Attenuation values in both phases were compared, and the leakage sign was considered positive if there was a greater than 10% increase in HU in the late-phase images compared to the arterial-phase images. The technical criteria are summarized in Fig. 1.

### Statistical Analysis

Data were analyzed using IBM SPSS Statistics for Windows (Version 26.0).

Initially, descriptive analyses of the baseline demographic and clinical-radiological variables were performed. Categorical variables were expressed as percentages, while quantitative variables were expressed as means or medians, depending on whether the distribution was parametric or non-parametric (normality was assessed using the Shapiro–Wilk test), along with their respective dispersion measures (standard deviation or interquartile range, respectively).

Univariate analysis was performed to study the association between various baseline variables and hematoma expansion as the primary outcome, as well as neurological deterioration and mortality as secondary outcomes. Odds ratios (OR), confidence intervals, and hypothesis tests (Chi-square or Fisher's exact test) were calculated for each variable. A p-value cutoff of ≤ 0.1 was used in the univariate analysis to select variables for inclusion in the multivariate analysis. This more flexible threshold, compared to the conventional p ≤ 0.05, was chosen to identify variables that may not be statistically significant on their own but could have an important influence when adjusted for other covariates in the multivariate model. Variables were selected using a forward stepwise method, with Wald statistics used as the criterion for inclusion.

Sensitivity and specificity values for the spot sign and leakage sign were calculated concerning their ability to predict hematoma expansion, neurological deterioration, and mortality.

Finally, a survival analysis was conducted using Kaplan–Meier curves for three independent groups:

1. Patients with a positive leakage sign,
2. Patients with a positive spot sign but negative leakage sign, and
3. Patients with both signs negative.

The log-rank test was used to compare survival curves, and the proportionality of risks was verified. Results with a p-value ≤ 0.05 were considered statistically significant.

## Results

The final sample included 94 patients who met the established inclusion criteria. The general characteristics of these patients are described in Table 1.

**Table 1:** Basal characteristics of the sample

Feature	n	%	Mean	SD
Sex				
M	63	67%	-	-
F	31	33%	-	-
Age	-	-	68,09	-
Location of hematoma				
Lobar	50	53,2%	-	-
Basal ganglia	44	46,8%	-	-
Brainsterm	0	0%	-	-
NIHSS at admision				
Anticoagulant therapy	19	20,2%		
Antiagregant theraphy	18	19,1%		
Previous HBP	63	67%		
SBP	-	-	178,98	37,61
DBP	-	-	96,89	22,50
Platelets	-	-	198827,96	68095,30
INR	-	-	1,37	0,80
aPTT	-	-	31,47	-
GCS	-	-	10,71	4,71

a. Qualitative variables were expressed as a percentage and continuous quantitative variables as a mean and its corresponding SD.

b. Abbreviations: NIHSS: National Institute of Health Stroke Score; HBP: High Blood Preasure; SBP: Systolic Blood Presure; DBP: Diastolic Blood Preasure; INR: International Normalized Ratio; aPTT: activated Partial Thromboplastin Time; GCS: Glasgow Coma Scale.

The average age of the patients was 68.09 years. It is also noteworthy that more than 67% of patients had various comorbidities unrelated to the hemorrhagic stroke episode, such as a prior diagnosis of hypertension.

### Analysis of the Outcome Variables

Hematoma expansion was observed in 42% of follow-up scans, neurological deterioration occurred in 15.5% of cases, and mortality was 39.4%. The predictive capacities of the various collected variables for these outcomes are presented in Table 2. The only factor independently associated with greater hematoma expansion [OR: 9.27 (95% CI: 2.95–29.20)], worse neurological deterioration [OR: 26.67 (95% CI: 1.62–47.39)], and mortality [OR: 7,56 (95% CI: 2,97–19,25)] was the presence of the leakage sign (see Table 3).

**Table 2:** Univariate of different baseline characteristics of the sample

Features	Univariate analysis								
	Hematoma Expansion			Neurological Impairment			In hospital Mortality		
	OR	95% CI	p	OR	95% CI	p	OR	95% CI	p
Sex									
M	1,69	0,58–4,94	0,33	3,20	0,37–28,01	0,29	1,28	0,52–3,11	0,59
Age	1,01	0,97–1,04	0,79	1,05	0,98–1,12	0,16	**1,04**	**1,01–1,08**	**0,01**
Location of Hematoma									
Lobar	**2,29**	**0,86–6,08**	**0,10**	3,96	0,75–20,99	0,11	1,52	0,66–3,51	0,33
NIHSS at admission	0,98	0,90–1,05	0,54	1,01	0,94–1,09	0,77	**1,18**	**1,07–1,30**	**0,01**
Anticoagulant Therapy	0,91	0,23–3,56	0,88	1,71	0,20–10,00	0,55	**4,08**	**1,37–12,14**	**0,01**
Antiagregant Therapy	**8,55**	**1,68–43,42**	**0,01**	1,11	0,2–6,21	0,90	1,98	0,72–5,46	0,19
History of HBP	1,94	0,69–5,42	0,21	5,07	0,59–43,79	0,14	**2,45**	**0,95–6,31**	**0,06**
SBP	**0,97**	**0,97–1,00**	**0,06**	0,99	0,97–1,01	0,43	1,00	0,99–1,01	0,78
DBP	0,98	0,96–1,00	0,15	0,9	0,97–1,02	0,40	0,99	0,97–1,01	0,25
Platelets	1,00	1,00–1,00	0,95	1,00	1,00–1,00	0,97	1,00	1,00–1,00	0,82
INR	1,29	0,44–3,75	0,65	**2,98**	**0,82–10,85**	**0,10**	**3,40**	**1,22–9,46**	**0,02**
aPTT	1,02	0,92–1,13	0,68	1,09	0,98–1,21	0,11	**1,06**	**1,00–1,13**	**0,06**
GCS	1,01	0,81–1,27	0,92	0,95	0,77–1,17	0,63	**0,63**	**0,50–0,80**	**0,00**
Initial Hematoma Volumen	0,99	0,98–1,01	0,31	0,97	0,94–1,01	0,11	1,00	0,99–1,01	0,76
Spot sign	**4,40**	**1,47–13,13**	**0,02**	**7,93**	**1,47–42,77**	**0,02**	**7,05**	**2,60–17,60**	**0,00**
Leakage Sign	**9,27**	**2,95–29,20**	**0,00**	**8,75**	**1,62–47,39**	**0,01**	**7,56**	**2,97–19,25**	**0,00**

**Bold** font was used to show variables associated with the different outcomes with p-value < 0.1, which were subsequently included in the multivariate study. Bold borders have been added to the cells of the two radiological signs under study

Abbreviations: NIHSS: National Institute of Health Stroke Score; HBP: High Blood Preasure; SBP: Systolic Blood Presure; DBP: Diastolic Blood Preasure; INR: International Normalized Ratio; aPTT: activated Partial Thromboplastin Time; GCS: Glasgow Coma Scale

**Table 3:** Multivariate analysis

	Features	Multivariate analysis (*)								
		Hematoma Expansion			Neurological Impairment			In hospital Mortality		
		OR	95% CI	p	OR	95% CI	p	OR	95% CI	p
	GCS	-			-			0,63	0,50–0,80	0,00
	Leakage Sign	9,27	2,95–29,2	0,00	26,67	1,62–47,39	0,01	7,56	2,97–19,25	0,00

(*) Only those variables that reached statistical significance (p-value < 0.05) are presented, following the stepwise selection process using the forward selection method based on the Wald statistic

Abbreviations: GCS: Glasgow Coma Scale

Another notable result is related to the 25 patients who did not undergo follow-up CT, of whom 8 died, with a median survival of 1.50 days (IQR: 2.00). Notably, all deceased patients exhibited a 100% prevalence of the leakage sign (p = 0.001). The remaining patients in this subgroup were discharged and subsequently transferred to other hospitals.

In the multivariate analysis, the only variables that were statistically significant were the leakage sign for hematoma expansion, neurological deterioration, and in-hospital mortality, as well as the GCS for neurological deterioration.

### Comparative Diagnostic Value of the Spot Sign and Leakage Sign

Both the spot sign and leakage sign demonstrated high specificity in predicting hematoma expansion and, to a lesser extent, mortality. Although the sensitivity values were lower, the leakage sign had notably higher values for predicting hematoma expansion.

Table 4 summarizes the sensitivity and specificity values for predicting hematoma expansion, in-hospital mortality, and neurological deterioration, considered as indirect indicators of hematoma expansion. The diagnostic performance of the spot sign and leakage sign is presented as percentages, accompanied by their respective confidence intervals (in parentheses).

**Table 4:** Analysis of the sensitivity and specificity values of the Spot Sign and the Leakage Sign

	Sensitivity			Specificity		
Features	Hematoma Expansion	Neurological Impairment	In hospital Mortality	Hematoma Expansion	Neurological Impairment	In hospital Mortality
Spot Sign	48,28% (29,45–67,47)	77,79% (39,99–97,19)	67,57% (50,22–81,99)	82,50% (67,22–92,46)	69,39% (54,59–81,75)	77,20% (64,16–87,26)
Leakage Sign	62,07% (42,26–79,31)	77,78% (39,99–97,19)	72, 97% (55,88–86,21)	85,00% (70,17–94,29)	71,43% (56,74–83,41)	73,68% (60,34–84,46

### Comparative Survival Analysis between the Spot Sign and the Addition of the Leakage Sign to the Diagnostic Scheme

**Fig. 2:**
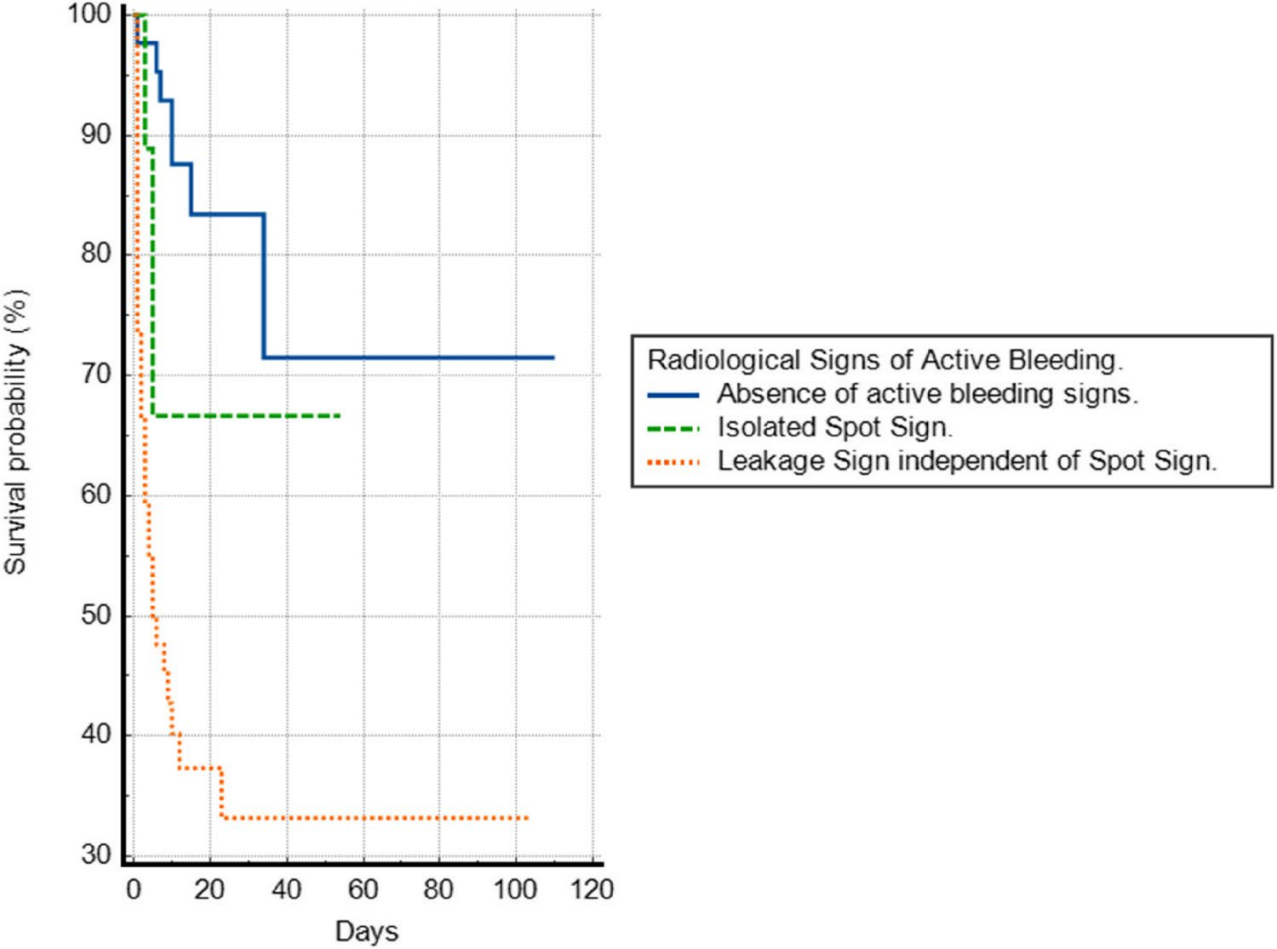
Survival graph showing the significant difference between the absence of active bleeding signs, isolated spot sign and the addition of the leakage sign in the diagnostic scheme

The median survival time was significantly lower (log-rank test < 0.001) in patients with a positive leakage sign (6 days) compared to those with an isolated positive spot sign (54 days) and patients with both signs negative (110 days). The survival curve is shown in Fig. 2.

## Discussion

This study aimed to analyze the utility of the spot sign and leakage sign as predictors of spontaneous intracerebral hematoma expansion. The spot sign has traditionally been recognized as a radiological marker that, after contrast administration, helps predict active bleeding within intracerebral hematomas. For instance, Wada et al. first described the spot sign in 2007 as a radiological feature capable of predicting active bleeding in cerebral hematomas [3, 18]. Later, in 2016, Orito et al. proposed the leakage sign as a variant for the same purpose, arguing that its addition increases sensitivity and specificity when combined with the spot sign, without introducing technical complications [16].

The leakage sign is relatively easy to identify and only requires a delayed imaging phase following contrast administration. This additional phase improves sensitivity and specificity for predicting hematoma growth. One of the limitations of the spot sign is that normal or abnormal vascular structures may be misinterpreted as points of active bleeding. The leakage sign can help differentiate these situations: if a leakage sign is negative in the presence of a positive spot sign, it is possible to rule out active bleeding and instead attribute the finding to a vascular structure [19–21].

A potential drawback of the leakage sign is the need for patients to remain in the CT room longer, which may pose a risk for patients with clinical instability and rapid deterioration. However, in our series, no patients experienced cardiorespiratory problems or other signs that would contraindicate the 5-min wait between contrast injection and image acquisition. Orito et al. [16] also reported waiting 5 min before performing the delayed-phase CT for the leakage sign, obtaining sensitivity (93.3% (95% CI: 0.757–0.988)) and specificity (88.9% (95% CI: 0.815–0.912)) values similar to those found in this study.

Multiple authors agree that sensitivity and specificity for predicting hematoma expansion are enhanced when there is a longer time interval between the non-contrast CT (NECT) and contrast-enhanced CT (CECT) [16, 20, 22, 23]. Rather than relying on the leakage sign, some studies have employed multiphase CTA protocols with more than two phases and varying time intervals between each phase. For instance, in a study on spot sign detection using multiphase CTA, David et al. [22] demonstrated that the likelihood of detecting contrast extravasation in patients with acute spontaneous intracerebral hemorrhage increased with later imaging phases. Specifically, they reported contrast extravasation in 29.3% of patients in the first phase (arterial), 43.1% in a second phase (4 s delayed), and 46.3% in a third phase (15 s delayed). The use of a two-phase protocol with a longer time interval, as in our study, reduces the possibility of technical confusion during the imaging process.

In contrast to previous studies based on multiphase CTA, which have focused on the spot sign, few publications describe a clear protocol for determining leakage sign positivity. In our study, we provided an objective and reproducible method for determining leakage sign positivity by introducing a percentage increase in attenuation (measured in Hounsfield units) in the delayed phase compared to the early phase after contrast administration.

Few studies have explored the relationship between the spot or leakage signs and neurological deterioration or mortality, focusing more on their association with hematoma expansion itself. In the series by Takwa et al. [24], 30% of the cohort showed hematoma expansion, which was associated with worse neurological outcomes (lower Glasgow Coma Scale and NIHSS scores) and a higher in-hospital mortality rate (53.6%) compared to those without hematoma expansion (6.3%). Similarly, a meta-analysis by Davis et al. [25] found that for every 10% increase in hematoma volume, there was a 5% increase in the risk of death and a 16% increase in the likelihood of a worse outcome on the modified Rankin Scale, as well as an 18% increase in the probability of transitioning from independent function to assisted living or poor performance on the Barthel Index.

Beyond its prognostic value, the leakage sign may have important clinical implications in the acute management of ICH. Its presence could prompt closer clinical and radiological monitoring, including repeat neuroimaging within 6–24 h or earlier neurosurgical consultation. In settings where expansion risk is high, the leakage sign could influence decisions regarding intensive care unit (ICU) admission, escalation of medical therapy (e.g., blood pressure management or reversal of anticoagulation), and even consideration of surgical interventions such as hematoma evacuation or decompressive craniectomy. While further research is needed to validate the impact of leakage sign–guided management strategies on outcomes, its high predictive value supports its integration into clinical decision-making pathways for high-risk ICH patients.

Notably, in our study, the spot sign and the leakage sign demonstrated comparable sensitivity and specificity for predicting early neurological deterioration. This contrasts with their respective performance in forecasting hematoma expansion and mortality, where the leakage sign consistently showed superior predictive accuracy. One possible explanation is that neurological worsening in acute ICH is multifactorial and not solely driven by hematoma growth. Factors such as baseline hematoma volume, location (e.g., thalamic or brainstem involvement), intraventricular extension, and systemic variables including age, anticoagulant use, and preexisting brain atrophy can all contribute to clinical decline. As a result, radiologic indicators of ongoing bleeding may not fully capture the pathophysiological complexity underlying neurological deterioration. Similar findings have been reported in prior studies, which demonstrated that predictors of hematoma expansion did not always align with clinical outcomes in the acute phase [26, 27]. These results emphasize the importance of combining imaging biomarkers with validated clinical scales to more accurately assess early neurological prognosis in patients with ICH.

Beyond its prognostic value, the leakage sign may also carry significant clinical implications in the acute management of ICH. Its early detection on initial imaging enables timely risk stratification and may justify a more proactive clinical approach. In practice, its presence could prompt intensive monitoring strategies, including closer neurological observation, repeat neuroimaging within 6–24 h, or expedited neurosurgical evaluation. Moreover, in patients at high risk of hematoma expansion, the leakage sign might influence key management decisions, such as admission to intensive care units, aggressive blood pressure control, or early reversal of anticoagulation. In selected cases, it could also support the indication for early surgical interventions, including hematoma evacuation or decompressive craniectomy. Although clinical protocols directly incorporating the leakage sign as a criterion for such interventions have yet to be standardized, Orito et al. [16] emphasized its high predictive value for hematoma expansion. Additionally, Morotti et al. [19], in their recent meta-analysis, proposed that the combined assessment of the spot and leakage signs improves prognostic accuracy and helps identify patients who may benefit from tailored therapeutic strategies. These findings underscore the potential of the leakage sign not only as a radiological marker of poor outcome but also as a tool to guide early, risk-adapted therapeutic interventions in high-risk ICH patients. Nonetheless, prospective studies are still needed to validate its impact on clinical outcomes and treatment efficacy.

### Limitations

Our study has several limitations. First, the implementation of a new protocol for this prospective study may have initially introduced technical difficulties in imaging or candidate selection. This issue was addressed through training of the involved professionals, ultimately resulting in a sample that met the established criteria. Second, the addition of a late-phase CT scan to the protocol increases the radiation dose received by the patient. However, this was validated by our hospital’s ethics committee, which deemed the potential clinical benefits outweighed the risks of additional radiation, especially for patients with potentially severe neurological conditions. In our series, the increase in radiation dose per patient was 670 DLP (dose-length product). Third, image review was performed in consensus by two radiologists. Future research should assess inter-observer variability through comparative studies.

Additionally, as this was a prospective study with a novel methodology not previously implemented in our hospital, there were instances where the on-call radiologists had no prior experience in performing or interpreting these studies. Nevertheless, the acquisition protocol was saved on the CT system for consistent use in all patients, and prior to the study's initiation, all on-call radiologists underwent basic training (2 h) conducted by two experienced neuroradiologists.

## Conclusion

In predicting spontaneous intracranial hematoma expansion, the evaluation of the leakage sign produced better results than the spot sign, with a notable increase in sensitivity and a slight increase in specificity. The presence of both signs was associated with neurological deterioration and in-hospital mortality, with the leakage sign showing a slightly stronger association.

## Data Availability

Data collected for analysis in this manuscript is available from the corresponding author upon reasonable request.
